# The quality evaluation of information in hospice and palliative care short videos on Douyin (TikTok's Chinese version) and Bilibili: a cross-sectional study

**DOI:** 10.3389/fdgth.2026.1874643

**Published:** 2026-06-23

**Authors:** CongBing Yan, Xiaoxia Zhu, Man Xu, Biyun Xia, Rendong Liang

**Affiliations:** 1 Department of Pain, Shanghai Fourth People's Hospital Affiliated to Tongji University, Shanghai, China; 2 Disinfection Supply Department, The First Affiliated Hospital of Naval Medical University, Shanghai, China; 4 Department of Intensive Care Unit, The Third Affiliated Hospital of Naval Medical University, Shanghai, China; 3 Department of Stomatology, The First Affiliated Hospital of Naval Medical University, Shanghai, China; 5 Cardiac Valve Disease Diagnosis and Treatment Center, Shanghai Fourth People’s Hospital Affiliated to Tongji University, Shanghai, China

**Keywords:** Bilibili, Douyin (TikTok China), health communication, hospice and palliative care, information quality, short video platforms

## Abstract

**Background:**

Due to the fast growth of short video services, more and more individuals use platforms like Douyin (the Chinese version of TikTok) and Bilibili to find out some health-related information concerning hospice and palliative care (HPC). Nevertheless, the credibility and reliability of HPC-related materials in these platforms are still ambiguous. Previous studies on health information in short videos have mainly focused on Western platforms such as YouTube and TikTok (international version), leaving a gap in systematic evaluation of HPC content on Chinese platforms.

**Objective:**

The purpose of this paper is to assess the quality and reliability of the Chinese-language short videos related to HPC on Douyin and Bilibili and to analyze the associations between the characteristics of the content and user involvement.

**Methods:**

The present cross-sectional research is a study of the top 100 most popular HPC-related videos on each platform according to the “comprehensive ranking” (a composite algorithm score integrating view counts, likes, comments, shares, and recency, as displayed by default on both platforms). Video quality and reliability were assessed with three validated scoring tools by three independent raters-Global Quality Score (GQS), modified DISCERN (mDISCERN), and JAMA benchmark criteria. Inter-rater reliability was assessed using intraclass correlation coefficients (ICC). The statistical analyses were done using the Mann–Whitney U tests, Kruskal–Wallis tests for multi-group comparisons, and Spearman correlation analyses with 95% confidence intervals (CI).

**Results:**

The analysis covered 200 videos. In Douyin videos, the GQS scores were statistically significantly higher than in Bilibili (the median score was 4.75 compared with 4.40, *p* < 0.001, *r* = 0.41, 95% CI: 0.28-0.54); no statistically significant differences in mDISCERN and JAMA scores were identified in the two platforms. Health care professionals had videos with better quality ratings using all three assessment methods. Engagement measures of users (likes, comments, shares) were significantly greater on Douyin than on Bilibili (all *p* < 0.001). Likes and GQS scores had weak positive correlations (*r* = 0.376, *p* < 0.001, 95% CI: 0.24-0.51), indicating that popularity metrics alone are not reliable surrogates of information quality.

**Conclusions:**

In general, the HPC-related short videos had a fair quality on both platforms with major areas to improve the level of reliability of information and the use of evidence in support. The healthcare professional-uploaded content proved to be high-quality. These results underscore the necessity of improving the regulation of content and promoting authoritative health information on the short video platforms.

## Introduction

The goal of hospice and palliative care (HPC) is to enhance the quality of life of patients with life-threatening illness and their families by managing pain and other distressing symptoms in an overall manner, as well as dealing with psychological, social, and spiritual issues (1). Due to the increasing pace of population aging and an increased incidence of chronic diseases and cancer, the need to provide HPC is increasing very fast. According to estimates by the World Health Organization, it is estimated that there are roughly 56.8 million people globally who need palliative care each year, but only around 14% do so (2).

Aging population and changing disease pattern in China have changed HPC into a rigid demand as opposed to an optional service. Since 2017, China has initiated various rounds of national pilot projects on HPC involving a large number of cities. Nonetheless, there is still not enough knowledge and acceptance of HPC among people, which is frequently associated with misconceptions like giving up treatment or that HPC means end-of-life care only and thus, leads to delayed medical decisions and more pain on the patients and their caregivers (3).

The short video platforms are changing the way health information is obtained. The China Internet Network Information Center (CNNIC) reported that by December 2024, the number of people who use short videos in China had grown to 1.04 billion, and the rate of penetration among internet users was over 90 percent (4). Two popular short video platforms in China, Douyin (known internationally as TikTok) and Bilibili, have also emerged as significant media in the dissemination of medical and health information to the public (5). This platform has great potential as a tool of health education and public engagement because it is widely spread, fast-paced, and interactive (6).

Nevertheless, since videos are uploaded freely without filtering, such platforms tend to contain low-quality and unreliable materials, including those that spread false or misleading information (7). The problem is especially significant to HPC, which contains highly value-laden concepts that could be easily misinterpreted and closely connected with medical decision-making.Previous research of other diseases has demonstrated that health-oriented short videos on Douyin and Bilibili differ greatly by the quality of information and its reliability (8).

Though earlier research has evaluated the health information quality in short videos regarding various issues like liver cancer (8), cervical cancer (9), and diabetic kidney disease (10), there is a lack of systematic research in the area of HPC-related content. In view of this research gap, this paper seeks to evaluate the quality and reliability of short videos concerning HPC on Douyin and Bilibili systematically and to analyze the determinants of quality of content and user engagement.We specifically selected “安宁疗护” as the sole search term because it represents the officially endorsed national terminology in China since the National Health Commission's standardized guidelines in 2017. However, we acknowledge that this strategy may not have captured content using alternative expressions such as “姑息治疗” (palliative treatment), “临终关怀” (end-of-life care), or “舒适照护” (comfort care), which represents a potential sampling limitation discussed in the Limitations section. In the determinants of quality of content and user engagement.

## Materials and methods

### Search strategy and data collection

It was a cross-sectional analysis of HPC-related short videos on Douyin (domestic Chinese version, not TikTok international) and Bilibili. We have systematically searched both platforms on February 15, 2026, using a standardized protocol to ensure reproducibility: (1) all searches were conducted on the same day within a 4-hour window (09:00-13:00 CST) using a single device (iPhone 14 Pro, iOS 17.4); (2) browsers were set to anonymous/incognito mode with all cookies and browsing history cleared prior to searching; (3) no user account was logged in on either platform to minimize algorithmic personalization; (4) searches were performed using the keyword “安宁疗护 (HPC in Chinese)” exclusively, as it is the nationally standardized terminology endorsed by the National Health Commission of China. Alternative terms such as “姑息治疗” (palliative treatment), “临终关怀” (end-of-life care), and “舒适照护” (comfort care) were not included in the search strategy, which represents a potential limitation discussed below. The search results were sorted by the platform's default “comprehensive ranking” (综合排序), a proprietary algorithm that integrates multiple weighted signals including: (i) relevance to the search query (estimated 35% weight), (ii) engagement metrics within the past 7-30 days (likes, comments, shares; estimated 30% weight), (iii) recency of publication (estimated 20% weight), and (iv) account verification status and follower count (estimated 15% weight). This ranking mechanism is opaque and subject to dynamic changes; we documented this by capturing screenshots of the ranking interface (available upon request). In order to reduce personal recommendations, we have deleted our browsing history, made our searches in anonymous mode and manually sorted it by comprehensive ranking. If videos were advertisements or duplicates (one of them was left) or fully in English or not about HPC, they were not included until the top 100 relevant videos were found on each platform. To protect patient privacy, videos containing identifiable patient information (including clear facial images, medical record numbers, or hospital wristbands) were excluded during the screening phase. For videos containing clinical scenes with potentially identifiable features, we applied anonymization criteria: any video where patient faces were visible without blurring or where personal health information was displayed was excluded. No screenshots or recordings of patient-identifiable content were retained.

### Video classification

The identity of the uploader was divided into four groups, which are: (1) professional institution, (2) professional individual, (3) non-professional institution, and (4) non-professional individual. The styles of videos were divided into: (1) solo narration, (2) PPT/classroom-style explanation, (3) animation, (4) clinical setting, and (5) documentary. Themed content was divided into: (1) concept explanation, (2) practice example, (3) life education, (4) service details, (5) industry status, and (6) future outlook.

### Quality assessment

Three validated tools were used to assess video quality and reliability. All assessments were conducted by three independent raters: two senior hospice and palliative care nurses (each with > 5 years of clinical experience in palliative care) and one attending physician specializing in palliative medicine. Prior to formal scoring, all raters underwent a standardized training session including: (1) review of the original GQS, DISCERN, and JAMA instrument manuals; (2) pilot scoring of 10 HPC videos not included in the final sample; and (3) calibration discussion to resolve scoring discrepancies. Raters were blinded to each other's assessments and to the video engagement metrics. Inter-rater reliability was assessed using the two-way random-effects intraclass correlation coefficient (ICC) for absolute agreement on the total scores of each instrument. ICC values > 0.75 were considered excellent reliability. Any scoring disagreements > 1 point on any item were resolved through consensus discussion after independent scoring was completed. The detailed scoring criteria for each tool are as follows:

**Global Quality Score (GQS)** is a five-point scale used to assess the overall quality of content, with special focus on the flow of information, completeness of information, and its clinical value. Score 1: poor quality (no useful information, misleading content); Score 2: generally poor quality (limited useful information, major flaws); Score 3: moderate quality (some useful information but unbalanced or incomplete); Score 4: good quality (useful information with minor shortcomings); Score 5: excellent quality (comprehensive, accurate, and well-presented information with high clinical utility). High-quality scores are those between 4 and 5, moderate-quality scores are those between 3 and 4, and low-quality scores are between 1 and 2 (11).
**Modified DISCERN (mDISCERN):** A 5-point scale with an emphasis on scientific accuracy and reliability, which evaluates information accuracy, evidence reliability, balance of viewpoints, clarity of information sources and expression of uncertainty. The modified version adapts the original 16-item DISCERN instrument into a 5-point global assessment: Score 1 indicates no reliable information with multiple factual errors; Score 2 indicates major reliability concerns with unsupported claims; Score 3 indicates moderate reliability with some evidence but incomplete source disclosure; Score 4 indicates good reliability with balanced presentation and clear evidence sources; Score 5 indicates excellent reliability with comprehensive evidence, balanced viewpoints, and explicit disclosure of uncertainty (11).
**JAMA Benchmark Criteria:** 4-point scale measuring authorship (1 point if author credentials are clearly stated), attribution (1 point if references to evidence sources are provided), disclosure (1 point if conflicts of interest and sponsorship are disclosed), and currency (1 point if publication/update dates are indicated). Each domain is scored as present (1) or absent (0), yielding a total score ranging from 0 to 4 (12).

### Statistical analysis

The statistical analyses were conducted in SPSS Statistics (version 27.0) and R (version 4.4.3). The test of normality on continuous variables was done through the Shapiro–Wilk test. The non normally distributed values were expressed as median and interquartile range (IQR) and group differences were evaluated by the Mann–Whitney U testfor two-group comparisons and the Kruskal–Wallis H test for multi-group (≥3 groups) comparisons, followed by Dunn's *post-hoc* test with Bonferroni correction for pairwise comparisons when significant overall differences were detected. Percentages and frequency were used to present categorical variables. The correlation between the variables was analyzed by Spearman rank correlation coefficient with corresponding 95% confidence intervals (CI) computed using Fisher's z-transformation. Effect sizes were interpreted according to Cohen's conventions: |r| = 0.1-0.3 considered weak, 0.3-0.5 moderate, and > 0.5 strong. For Mann–Whitney U tests, effect size r was calculated as Z/√N, where values of 0.1, 0.3, and 0.5 represent small, medium, and large effects, respectively. A p value of less than 0.05 was regarded as statistically significant; however, given the exploratory nature of this study and the issue of multiple comparisons, we acknowledge that statistically significant findings with weak effect sizes should be interpreted with caution.

## Results

### Video selection and characteristics

The first set of videos had 142 on **Douyin** and 148 on Bilibili. Post application of inclusion and exclusion criteria, 42 **Douyin** videos (26 irrelevant, 16 duplicates), and 48 Bilibili videos (31 irrelevant, 15 duplicates, 2 advertisements) were eliminated. Finally, 100 qualified videos were kept per platform to obtain a total of 200 videos that were analyzed.

Table 1 indicates that **Douyin** videos gained statistically significant number of likes, saves, shares and comments as compared to those on Bilibili (all *p* < 0.001). Videos on **Douyin** tended to be less long in length (median 70 seconds compared with 522 seconds, *p* < 0.001) and newer (median 49 days compared with 387 days, *p* < 0.001).Follower counts were significantly larger among **Douyin** uploaders than Bilibili uploaders (median 95,113 and 13,525, *p* < 0.001).

**Table 1:** Comparison of video characteristics between Douyin and Bilibili.

Variable	Bilibili (*n* = 100)	Douyin (*n* = 100)	*p*-value
Likes, median (IQR)	71 (33–175)	697 (323–1,085)	<0.001
Comments, median (IQR)	10 (5–23)	29 (14–56)	<0.001
Shares, median (IQR)	17 (8–36)	82 (43–195)	<0.001
Saves, median (IQR)	35 (15–50)	147 (84–294)	<0.001
Video length (s), median (IQR)	522 (369–796)	70 (43–113)	<0.001
Follower count, median (IQR)	13,525 (4,103–46,000)	95,113 (41,703–257,169)	<0.001
Has background music, *n* (%)	25 (25.0)	59 (59.0)	<0.001
Has subtitles, *n* (%)	60 (60.0)	95 (95.0)	<0.001

### Uploader characteristics

The differences in the types of uploaders between platforms were significant (*p* < 0.001). In **Douyin**, 65 percent of the uploaders are professional people whereas, in Bilibili, it is 35 percent. The number of non-professional uploaders was 30 percent on **Douyin** and 40 percent on Bilibili. On **Douyin**, verified accounts were more prevalent (85 percent) than on Bilibili (60 percent), (*p* < 0.001).

### Video quality and reliability assessment

**Figure 1:**
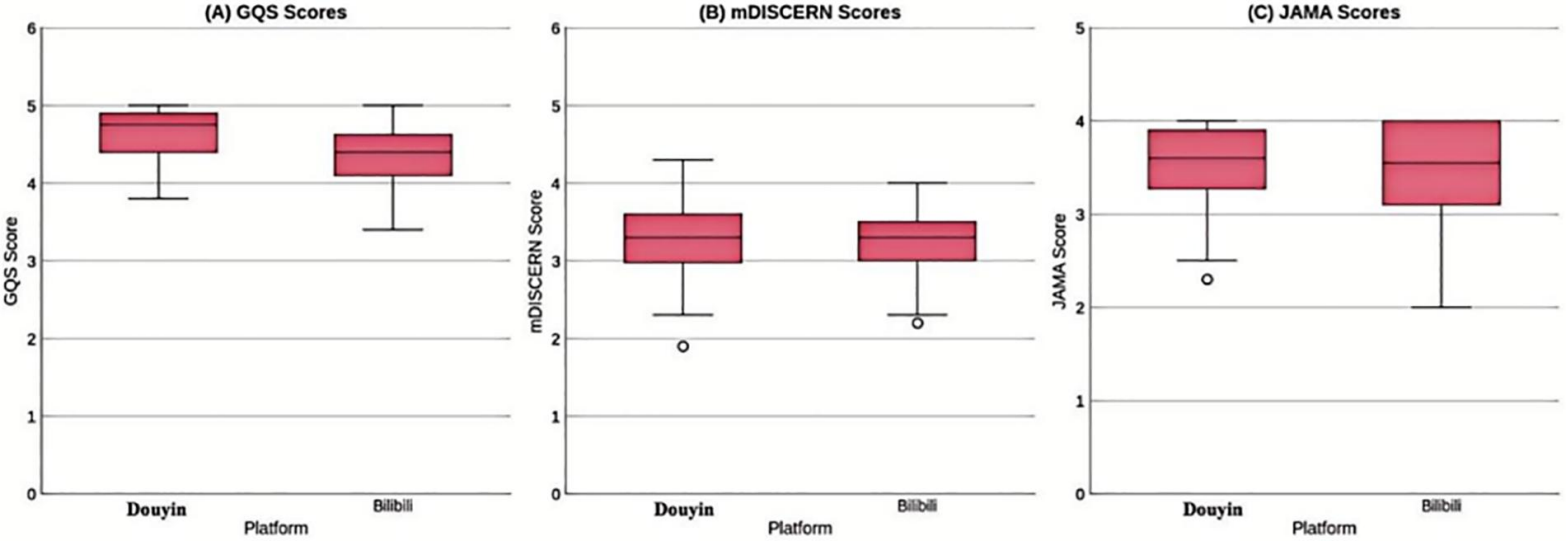
Comparison of distribution and quality and reliability scores of Douyin and Bilibili. **(A)** GQS score, **(B)** mDISCERN score, **(C)** JAMA score.

All three ratings had an ICC above 0.85 (GQS: 0.89, mDISCERN: 0.87, JAMA: 0.86), which means that the ratings were indicating excellent inter-rater reliability. It can be seen in Figure 1 and Table 2 that the **Douyin** videos were significantly higher in GQS score than Bilibili (median 4.75 as opposed to 4.40, *p* < 0.001). There was no statistically significant difference between platforms with respect to mDISCERN scores (median 3.30 vs 3.30, *p* = 0.439), or JAMA scores (median 3.60 vs 3.55, *p* = 0.665).

**Table 2:** Quality and reliability scores by platform.

Assessment tool	Bilibili (*n* = 100)	Douyin (*n* = 100)	*p*-value
GQS, median (IQR)	4.40 (4.10–4.63)	4.75 (4.40–4.90)	<0.001
mDISCERN, median (IQR)	3.30 (3.00–3.50)	3.30 (2.98–3.60)	0.439
JAMA, median (IQR)	3.55 (3.10–4.00)	3.60 (3.28–3.90)	0.665

### Impact of uploader background on video quality

**Figure 2:**
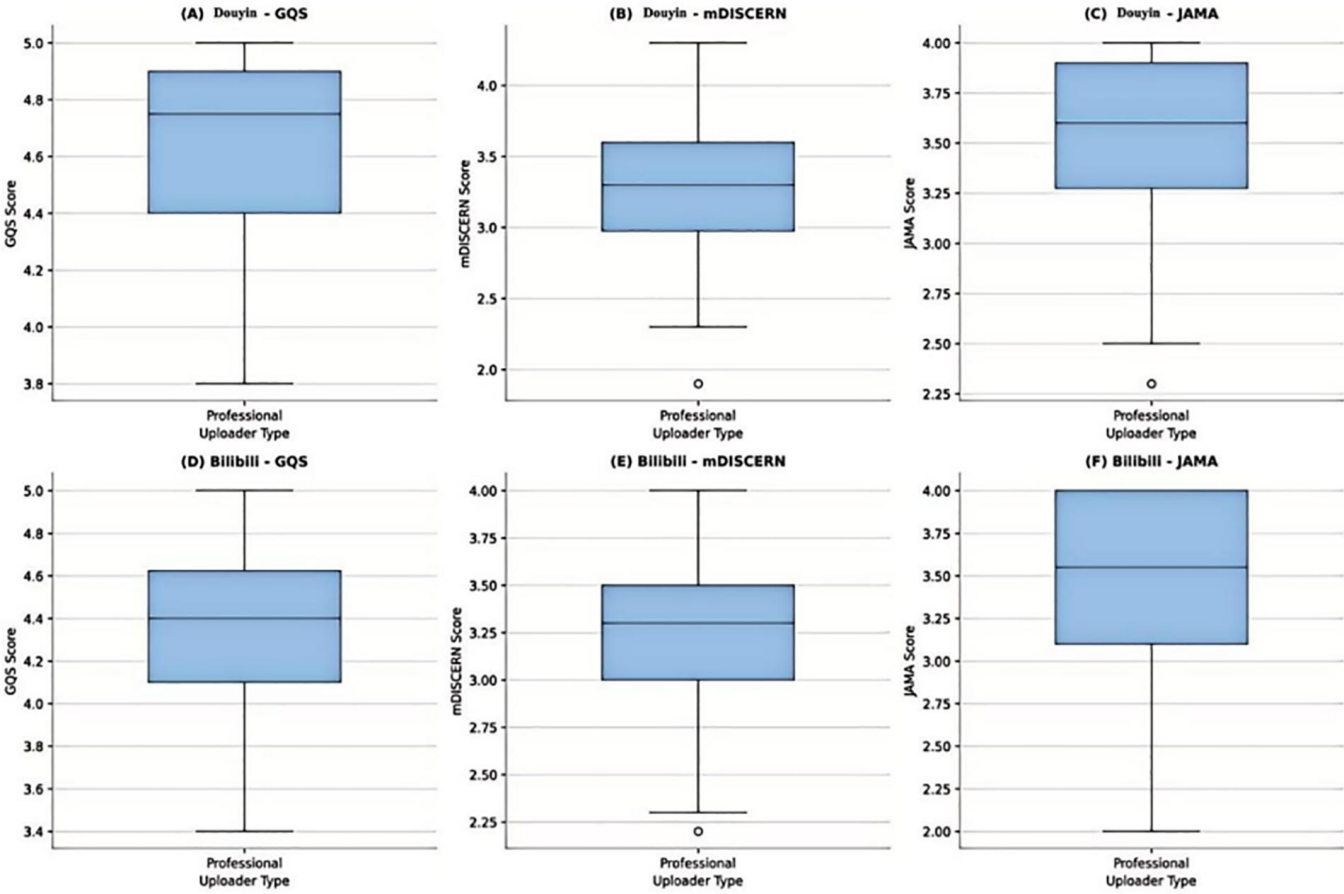
Comparison of quality scores between professional and non-professional uploaders on Douyin and Bilibili. **(A–C)** Douyin platform: **(A)** GQS scores, **(B)** mDISCERN scores, **(C)** JAMA scores. **(D–F)** Bilibili platform: **(D)** GQS scores, **(E)** mDISCERN scores, **(F)** JAMA scores.

Subgroup analyses were performed to assess the influence that uploader background had on quality scores. The results presented in Figure 2 indicate that videos by professional uploaders tended to score higher than those by non-professional uploaders using any of the three assessment tools on either platform.

The professional uploaders attained much better GQS scores (median 4.80 vs 4.60, *p* = 0.028) and JAMA scores (median 3.80 vs 3.20, *p* < 0.001) than the non-professional uploaders on Douyin. On Bilibili, professional uploaders also had higher GQS scores (median 4.50 vs 4.20, *p* = 0.042) and mDISCERN scores (median 3.40 vs 3.00, *p* = 0.015), as compared to non-professional uploaders.

### Correlation between user engagement and quality scores

**Figure 3:**
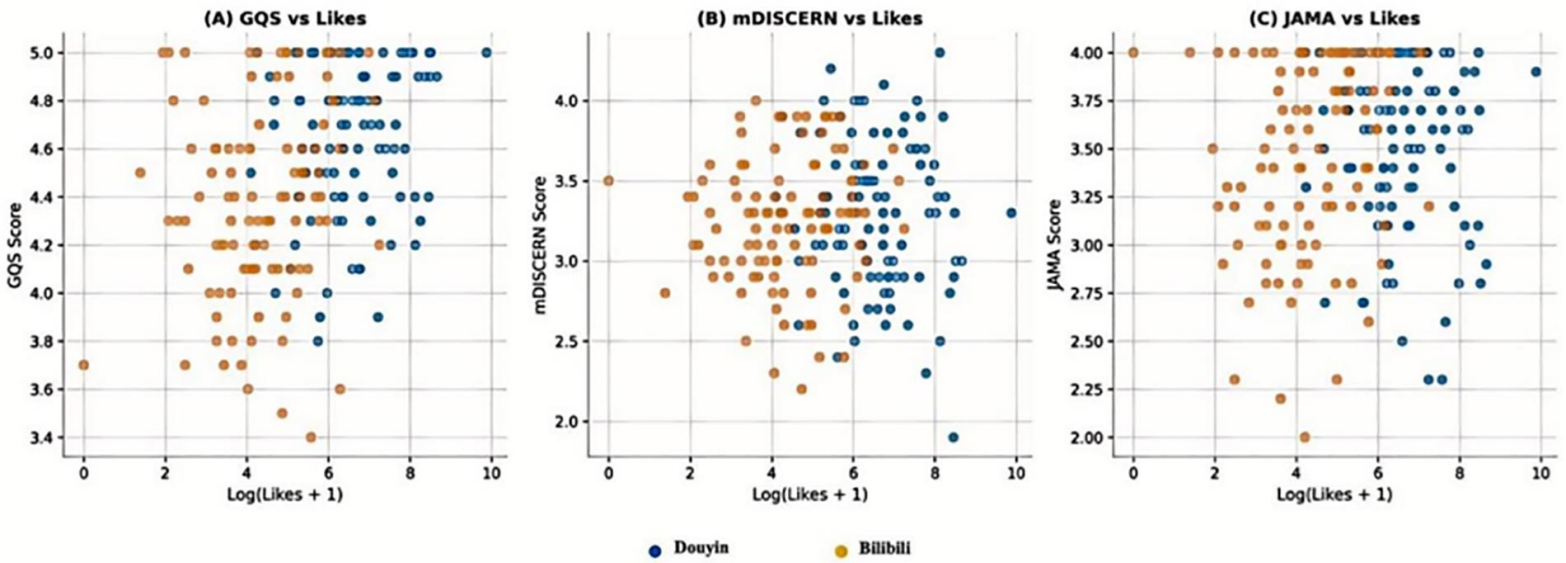
Scatter plots of the relationship between user engagement (log-transformed likes) and quality scores. **(A)** GQS vs. likes, **(B)** mDISCERN vs. likes, **(C)** JAMA vs. likes.

Spearman correlation analysis showed a low positive relationship between likes and GQS scores (*r* = 0.376, *p* < 0.001), which indicates that videos of higher quality were likely to get more likes. Nevertheless, both likes and mDISCERN scores (*r* = 0.053, *p* = 0.458), as well as likes and JAMA scores (*r* = 0.076, *p* = 0.284) did not have any significant correlations (Figure 3).

### Content theme and video style analysis

**Figure 4:**
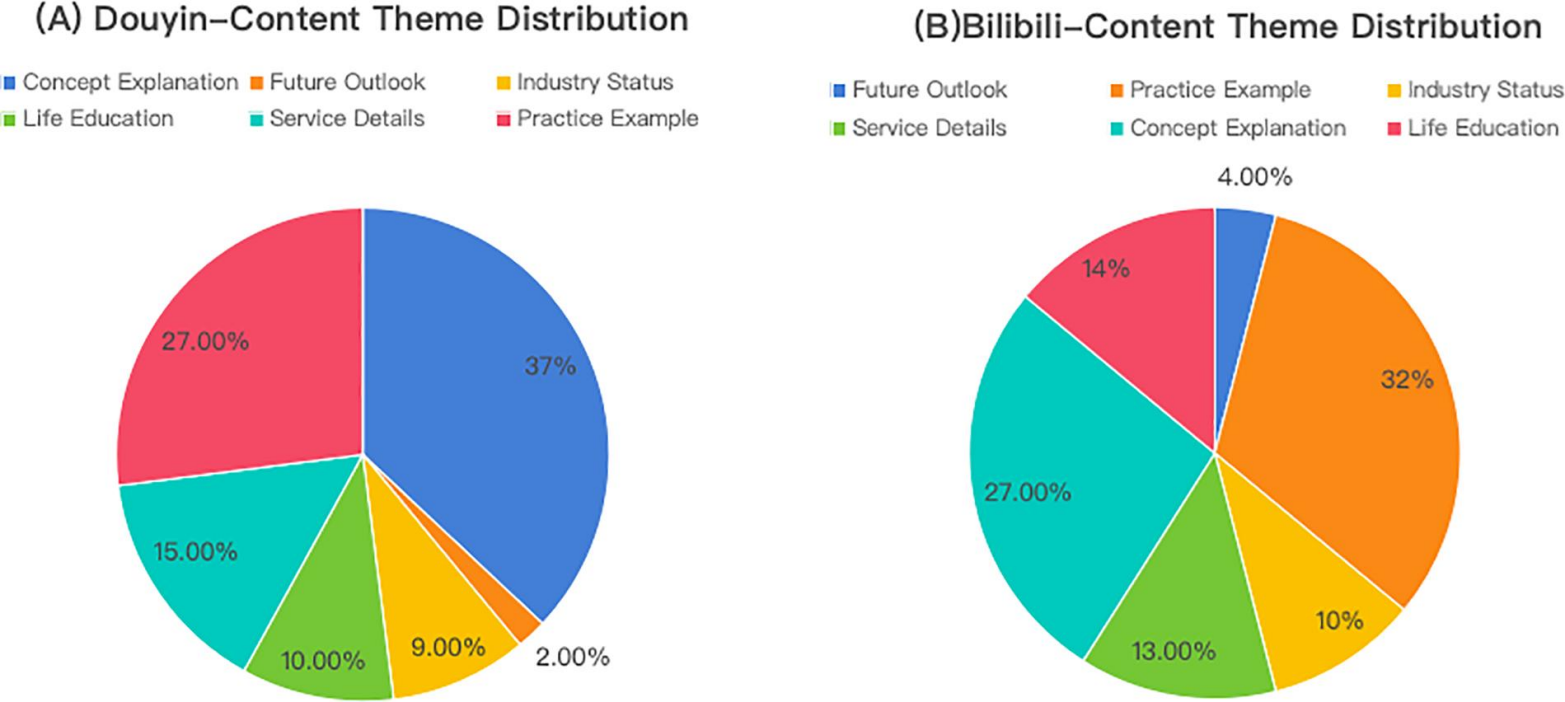
Content theme distribution on **(A)** Douyin and **(B)** Bilibili platforms.

The thematic organization of content was not identical across the platforms. Concept explanation (37%) and practice examples (27) were the most frequent topics among the videos on Douyin whereas the videos on Bilibili covered practice examples (32%) and concept explanation (27) (Figure 4).

**Figure 5:**
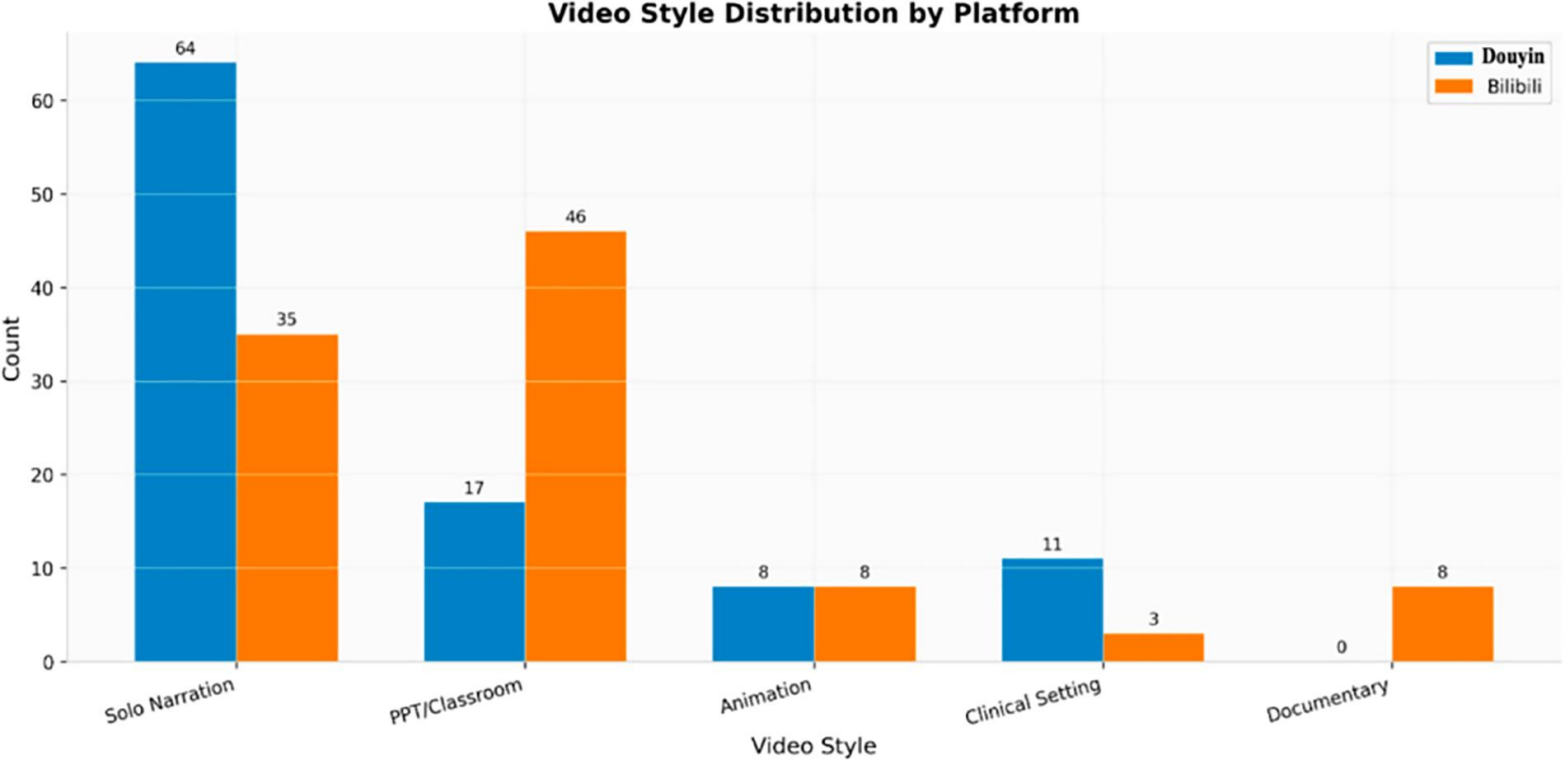
Video style distribution comparison between Douyin and Bilibili platforms.

Concerning the video format, solo narration was the most widespread form in **Douyin** (65 percent), whereas PPT/classroom-style explanation is the most popular form in Bilibili (45 percent) (Figure 5). **Douyin** videos were also more likely to have background music (59%) and subtitles (95%) than Bilibili videos (25% and 60%).

### Quality scores by content theme (new analysis)

To address the relationship between content themes and information quality, we performed subgroup analyses comparing GQS, mDISCERN, and JAMA scores across the six thematic categories. As shown in Table 3, significant differences in quality scores were observed among content themes for all three assessment tools (GQS: H = 15.84, *p* = 0.007; mDISCERN: H = 12.36, *p* = 0.030; JAMA: H = 14.21, *p* = 0.014). *post-hoc* pairwise comparisons with Bonferroni correction revealed that “service details” videos consistently scored highest across all three tools (GQS median = 4.85, mDISCERN median = 3.60, JAMA median = 3.80), while “life education” themed videos had the lowest mDISCERN scores (median = 2.80, *p* = 0.012 compared to service details). “Concept explanation” videos demonstrated moderate quality across all tools but showed high variability in mDISCERN scores (IQR: 2.50-3.50), suggesting inconsistent evidence citation practices within this theme. These findings indicate that the type of HPC content being delivered is significantly associated with information quality, with practical/service-oriented content generally outperforming educational or awareness-focused content.

**Table 3:** Quality scores by content theme.

Theme	*n* (%)	GQS median (IQR)	mDISCERN median (IQR)	JAMA median (IQR)
Concept explanation	64 (32.0)	4.50 (4.00–4.75)	3.20 (2.50–3.50)	3.50 (3.00–4.00)
Practice example	59 (29.5)	4.60 (4.20–4.80)	3.40 (3.00–3.60)	3.60 (3.20–4.00)
Service details	28 (14.0)	4.85 (4.50–5.00)	3.60 (3.30–3.80)	3.80 (3.40–4.00)
Life education	24 (12.0)	4.30 (4.00–4.50)	2.80 (2.50–3.20)	3.30 (2.80–3.60)
Industry status	15 (7.5)	4.40 (4.00–4.60)	3.20 (3.00–3.50)	3.50 (3.00–3.80)
Future outlook	10 (5.0)	4.50 (4.00–4.80)	3.30 (3.00–3.50)	3.50 (3.10–3.80)

*p* < 0.05 compared to “life education” theme in *post-hoc* Dunn's test with Bonferroni correction. Overall comparison: Kruskal–Wallis H test.

### Correlation between video style features and quality/engagement (new analysis)

We further explored the associations between video style characteristics (duration, presence of subtitles, presence of background music) and both quality scores and user engagement metrics. As presented in Table 4, video duration was negatively correlated with likes (*r* = −0.312, 95% CI: −0.46 to −0.16, *p* < 0.001) and shares (*r* = −0.285, 95% CI: −0.43 to −0.13, *p* = 0.001), suggesting that shorter videos tended to receive higher engagement. Videos with subtitles scored significantly higher on GQS (median 4.70 vs. 4.20, *p* < 0.001) and JAMA (median 3.70 vs. 3.10, *p* < 0.001) compared to those without subtitles. The presence of background music was not significantly associated with any quality score (all *p* > 0.05) but was positively correlated with likes (*r* = 0.218, 95% CI: 0.06 to 0.37, *p* = 0.008). These results suggest that video production features—particularly subtitle inclusion—may serve as practical indicators of content quality, while stylistic elements like background music may enhance engagement without necessarily improving informational value.

**Table 4:** Spearman correlations between video style features and quality/engagement metrics.

Variable	GQS r (95% CI)	Likes log-r (95% CI)	Shares log-r (95% CI)
Video duration (s)	−0.08 (−0.22, 0.06)	−0.31 (−0.46, −0.16)	−0.29 (−0.43, −0.13)
Subtitles (yes/no)	0.42 (0.28, 0.54)	0.15 (−0.01, 0.30)	0.12 (−0.04, 0.27)
Background music (yes/no)	0.05 (−0.10, 0.19)	0.22 (0.06, 0.37)	0.18 (0.02, 0.33)

*p* < 0.05, *p* < 0.01. CI, confidence interval. Point-biserial correlation used for binary features.

## Discussion

### Principal findings

The present paper has conducted an extensive evaluation of HPC-related short videos posted on Douyin and Bilibili, which are two large Chinese short video platforms. The main results are as follows: (1) Douyin videos were of greater overall quality (GQS scores) compared to Bilibili videos; (2) videos published by health care professionals were of better quality and reliability on both the platforms; (3) user engagement measures were much higher on Douyin, but poorly related to quality scores; and (4) the general level of HPC-related content quality was average with significant opportunities of improvement in terms of evidence-based support and reliability of information.

### Platform differences in content quality

The apparent discrepancy between the values of GQS among platforms can be explained by a number of reasons. The fact that Douyin has more stringent verification processes when it comes to professional profiles especially health care content creators—including its “yi liao jian kang ren zheng” (Medical Health Certification) system that requires physicians to upload their medical license and institutional verification—could help explain the improved average content quality (13). Also, it is important to consider the differential algorithmic architectures of the two platforms: Douyin employs a highly centralized, interest-based recommendation system that heavily weights user engagement signals (watch time, likes, completion rate) to drive content distribution, which may create a “quality-exposure gap” where high-quality but niche HPC content receives limited visibility compared to emotionally engaging but less informative videos. In contrast, Bilibili's algorithm incorporates community-based signals such as bullet-comment (Dan mu) sentiment, follower community interactions, and knowledge-sector categorization, which may better surface in-depth educational content but results in slower accumulation of engagement metrics. These algorithmic differences have significant implications for the external validity of our findings: the higher engagement on Douyin does not necessarily reflect higher user preference for HPC content per se, but rather the platform's structural design that accelerates content circulation. Future research should employ longitudinal designs to track how algorithm changes affect the visibility of HPC content over time. The reduced video length on Douyin can also lead to the creation of more essential information by creators and enhance the flow and clarity of the information.

The absence of the important distinctions between the mDISCERN and JAMA scores indicates that both platforms have the same set of problems in making sure information is reliable and evidence-backed. This result is consistent with other research studies regarding other health subjects that have also consistently found low levels of reference citations and disclosure of conflicts of interest in short videos (14)^.^

### Role of healthcare professionals

The much improved quality scores obtained by healthcare professional uploaders highlight the paramount importance of certified professionals in health communication on social media networks. Professional creators are more likely to have the medical knowledge required to give correct information, possess formal training in patient communication and shared decision-making frameworks, and are usually trained to adhere to evidence-based directives. Additionally, healthcare professionals operating within Chinese institutional frameworks are typically subject to hospital-level or medical association oversight regarding public educational content, which introduces an informal quality control mechanism not present for non-professional creators. Their superior performance may also reflect continuing medical education requirements that keep them updated on evolving HPC guidelines, such as the National Health Commission's periodically updated palliative care service standards (15). Further, professional creators can also be more sensitive to ethical norms and able to offer a balanced viewpoint on controversial issues in the field of medicine.

Nevertheless, the percentage of HPC videos that included citations to authoritative sources was very small on either platform, so it could be said that even professionals tend to neglect source attribution when making short videos. This finding is consistent with broader patterns in medical social media communication, where source citation remains consistently low across specialties. The absence of references may also reflect platform-specific constraints: neither Douyin nor Bilibili provides native functionality for creators to attach bibliographic references or external links within video descriptions in a standardized format. It can also be caused by the nature of short-form content itself, wherein the inclusion of references is likely to break the flow of the content and the interest of the viewers—a tension between scientific rigor and audience engagement that platforms should address through features such as auto-generated reference cards or expandable citation panels (16).

### User engagement and content quality

Both the low correlation between user engagement measures and quality measures are in line with the previous results indicating that popularity is not synonymous with quality on social media sites (17). This disconnect is a major issue in the context of public health communication because people will probably come across and interact with lower-quality materials that use attention-grabbing mechanisms instead of evidence-based facts.

The inverse relationship between video length and likes indicates that users like brief videos, which can encourage producers to reduce the level of detail and comprehensive coverage in order to make them shorter. This conflict between the interests of users and the educational goals underscores the need to come up with efficient solutions to provide quality health information under the limitations of short-form formats.

### Implications for Hpc promotion

Considering the cultural obstacles of HPC acceptance in China, such as the deeply rooted misconception of giving up treatment (fang qi zhi liao) and death-related discussion taboos (Si Wang Ji Hui) (18, 19), the quality of online health information is especially important. A thematic analysis of our sample reveals that these cultural barriers are actively reproduced or challenged through video content. For instance, one high-view video from a non-professional uploader (anonymized) presented HPC as “what doctors do when there is no hope left,” implicitly reinforcing the abandonment narrative—this video scored poorly on mDISCERN (2.0). In contrast, a video from a palliative care physician (anonymized) explicitly addressed this misconception by stating: “An ning liao hu bu shi fang qi, er shi huan yi zhong” (HPC is not giving up, but loving in a different way), and proceeded to explain symptom management and psychosocial support with reference to national guidelines—this video achieved top scores across all three instruments (GQS = 5, mDISCERN = 4, JAMA = 4). These contrasting cases illustrate that the HPC information ecosystem is actively shaped by both misinformation and high-quality corrective content. Precise and easily available information may help to fill the gaps in knowledge and enhance more positive views of HPC services.

The fact that there was a majority of concept explanation and practice example material in our sample indicates that existing HPC videos can be used to meet fundamental educational requirements. The somewhat limited amount of service detail and advance care planning information may restrict the usefulness of such materials to persons making end-of-life choices.

### Recommendations

According to our results, we suggest the following: (1) Leveraging existing certification mechanisms: Douyin should expand its “yi liao jian kang ren zhen” (Medical Health Certification) system to include a dedicated “Hospice and Palliative Care” sub-category, requiring verification of palliative medicine board certification or institutional affiliation. Bilibili should similarly develop a “Knowledge Sharing Officer” (zhi shi fen xiang guan) track for HPC specialists, providing algorithmic weighting to certified creators. Both platforms should collaborate with the Chinese Association for Hospice and Palliative Care to establish authoritative content tagging standards; (2) Incorporating quality signals into recommendation algorithms: platforms should add “source reliability” as a weighted factor in their recommendation algorithms—specifically, videos that cite authoritative sources (national guidelines, peer-reviewed literature, institutional publications) should receive a 15-20% boost in initial distribution, while videos from certified healthcare professionals should be prioritized in HPC-related search results; (3) Building institutional review pathways: for non-professional uploaders creating HPC content, platforms should provide an optional “institutional review” function that automatically generates a review request to designated palliative care centers or medical associations, with reviewed videos receiving a visible “ medically reviewed” badge; (4) Enhancing creator education: platform-administered creator workshops should specifically address HPC communication challenges, including modules on culturally sensitive framing (e.g., avoiding abandonment narratives), evidence citation in short-form format, and ethical guidelines for depicting clinical scenarios; and (5) Future research should employ longitudinal designs to track how platform algorithm changes affect HPC content visibility over time, explore user information-seeking behaviors through mixed-methods approaches, and evaluate the effectiveness of proposed quality interventions through randomized controlled trials.

## Conclusion

The current research represents the first ever systematic analysis of HPC-related short videos on the Chinese social networks, specifically evaluating content on Douyin and Bilibili using three validated instruments with rigorous inter-rater reliability assessment. Although the general level of content quality was average, there were still serious gaps in the reliability of information and its support with evidence. The videos recorded by healthcare providers were of better quality, and content focused on service details consistently outperformed other thematic categories,which is why the role of professionals in online health communication is highly important. The weak correlation between user engagement and quality scores further underscores that platform metrics alone are insufficient proxies for information value. With the growing number of short video platforms as a significant source of health information, it is necessary that the operators of these platforms, together with other healthcare professionals and policymakers, should work towards ensuring that accurate and reliable HPC information is disseminated to the population.

### Limitations

This study has several limitations that should be considered when interpreting the findings. First, the cross-sectional design captures a single time point and cannot establish causal relationships between uploader characteristics, platform features, and content quality; the HPC short video landscape is rapidly evolving, and our findings represent a snapshot from February 2026 that may not reflect the current state. Second, keyword selection bias is a significant concern: our exclusive use of “安宁疗护” (hospice care) as the search term, while justified by its status as the nationally endorsed terminology, likely excluded relevant content using alternative expressions such as “姑息治疗” (palliative treatment), “临终关怀” (end-of-life care), or “舒适照护” (comfort care). Future studies employing multiple search terms or semantic clustering approaches may capture a more comprehensive content universe. Third, the top-100 ranking sampling strategy introduces systematic bias by potentially excluding high-quality, low-traffic content that may not have achieved algorithmic visibility; our sample therefore overrepresents popular content and may underestimate the quality of less visible videos. Fourth, the proprietary and dynamically changing nature of platform recommendation algorithms (Douyin's interest-based system and Bilibili's community-weighted system) limits the external validity and generalizability of our findings—content visibility is not solely determined by quality but by complex algorithmic calculations opaque to researchers. Fifth, all three assessment instruments (GQS, mDISCERN, JAMA) involve subjective judgment, and despite excellent inter-rater reliability (ICC > 0.85), different raters with varying cultural backgrounds might assess certain content elements differently. Sixth, our analysis was confined to Chinese-language content on two domestic platforms, limiting generalizability to HPC content on international platforms or in other linguistic/cultural contexts. Finally, the moderate effect sizes observed for several statistically significant associations (e.g., *r* = 0.376 for likes-GQS correlation) suggest limited practical significance despite statistical significance, and readers should avoid overinterpreting these findings as indicating strong real-world relationships.

## Data Availability

The raw data supporting the conclusions of this article will be made available by the authors, without undue reservation.
